# Results of an online questionnaire to survey calf management practices on dairy cattle breeding farms in Austria and to estimate differences in disease incidences depending on farm structure and management practices

**DOI:** 10.1186/s13028-015-0134-y

**Published:** 2015-08-19

**Authors:** Daniela Klein-Jöbstl, Tim Arnholdt, Franz Sturmlechner, Michael Iwersen, Marc Drillich

**Affiliations:** Clinical Unit for Herd Health Management in Ruminants, University Clinic for Ruminants, Department for Farm Animals and Veterinary Public Health, University of Veterinary Medicine Vienna, Veterinaerplatz 1, 1210 Vienna, Austria; Association of Austrian Cattle Breeders (ZAR), Dresdner Str. 89/19, 1200 Vienna, Austria

**Keywords:** Questionnaire, Dairy calf, Management, Diarrhoea

## Abstract

**Background:**

Calf disease may result in great economic losses. To implement prevention strategies it is important to gain information on management and to point out risk factors. The objective of this internet based survey was to describe calf management practices on registered dairy breeding farms in Austria and to estimate differences in calf disease incidences depending on farm structure and management practices.

**Results:**

A total of 1287 questionnaires were finally analysed (response rate 12.2 %). Herd characteristics and regional distribution of farms indicated that this survey gives a good overview on calf management practices on registered dairy farms in Austria. The median number of cows per farm was 20 (interquartile range 13–30). Significant differences regarding farm characteristics and calf management between small and large farms (≤20 vs >20 cows) were present. Only 2.8 % of farmers tested first colostrum quality by use of a hydrometer. Storing frozen colostrum was more prevalent on large farms (80.8 vs 64.2 %). On 85.1 % of the farms, whole milk, including waste milk, was fed to the calves. Milk replacer and waste milk were more often used on large farms. In accordance with similar studies from other countries, calf diarrhoea was indicated as the most prevalent disease. Multivariable logistic regression analysis revealed that herd size was associated with calf diarrhoea and calf respiratory tract disease, with higher risk of disease on large farms. Furthermore, feeding waste milk to the calves was associated with increasing calf diarrhoea incidence on farm. In the final model with calf respiratory tract disease as outcome, respondents from organic farms reported less often a respiratory tract disease incidence of over 10 % compared with conventional farms [odds ratio (OR) 0.40, 95 % confidence interval (CI) 0.21–0.75] and farmers that housed calves individually or in groups after birth significantly reported more often to have an incidence of respiratory tract disease >10 % compared with farms where all calves were housed individually (OR 2.28, 95 % CI 1.16–4.48).

**Conclusion:**

The results obtained in this study provide an overview on calf management on dairy breeding farms in Austria and may help to further point out areas to be improved on farm.

**Electronic supplementary material:**

The online version of this article (doi:10.1186/s13028-015-0134-y) contains supplementary material, which is available to authorized users.

## Background

Calf morbidity and mortality result in great economic losses [1, 2]. Therefore, it is of importance to find optimal intervention and prevention strategies to reduce the risk of calf diseases on farms [3]. Diarrhoea and respiratory tract disease are the most frequent diseases in calves [4, 5]. Several risk factors for calf diseases, particularly diarrhoea, have been identified, including farm size, presence of a calving pen and hygiene in this area, the quality of colostrum and the route of colostrum feeding as well as type of calf housing [4, 6–9].

Calf management has been evaluated in several European countries and in North America with larger cattle herds (mean >40 cows) [10–13]. In Austria, dairy farms are traditionally family-owned and small sized with an average number of 18 dairy cows, predominantly Fleckvieh breed [14]. Therefore, it can be assumed that the calf management on these small farms, similarly to countries with comparable agricultural structures, differs from that of other countries. Also, calves of different breeds might be managed differently as reported by Stanek et al. [13] for Holstein–Friesian and Fleckvieh calves in Czech Republic.

The objective of the present study was to describe calf management practices on registered dairy breeding farms in Austria and to estimate differences in disease incidences depending on farm structure and management practices.

## Methods

### Study population

Approximately 10,500 cattle breeders registered in the Association of Austrian cattle breeders (ZAR) were chosen as study population. Registered farms cover 66 % of all dairy farms and 78 % of all dairy cows in Austria [14].

### Questionnaire

An internet-based questionnaire was designed by using Google Forms [15]. The questionnaire comprised five areas of interest: (1) farm characteristics, (2) calving and care of the newborn, (3) calf housing, (4) calf feeding, and (5) calf disease and mortality in pre-weaned calves. In total, the questionnaire consisted of 32 questions, with supplementary questions depending on the given answers. The questions were semi-closed, closed, multiple choice, and open questions. The questionnaire was tested by ten selected farmers for comprehensibility and clarity before the hyperlink was sent out via email to all registered dairy breeders. The survey was online for 4 weeks from October to November 2012. Farmers could answer the questionnaire anonymously. The questionnaire (in German) is provided as Additional file 1.

### Data analyses

All records were edited by individually examining for aberrant results and plausibility before statistical analyses. Questionnaires with less than five answers given in total in area of interest 2 to 5 (2) calving and care of the newborn, (3) calf housing, (4) calf feeding, and (5) calf disease and mortality were excluded from statistical analyses. Free text statements were assessed individually and grouped according to their similarity where appropriate.

Data were statistically analysed using PASW, version 20.0 (IBM Cooperation, New York, USA). Descriptive statistics were calculated for farm characteristics and management practices.

For a more detailed analysis, the farms were categorised by herd size, farmers’ reported incidence of diarrhoea and respiratory tract disease (within 1 year). Herd size was categorised according to the median number of cows on farm (≤20 vs >20 cows). Differences between small and large herds were evaluated by use of Chi square tests.

To facilitate the farmers the questions regarding disease incidence, they were categorised with the answers ≤10 %, 11–25 %, 26–50 %, 51–75 %, and >75 %. For further analysis, farms were categorised into farms with a maximum of 10 % and with more than 10 % of calves affected by diarrhoea or respiratory tract disease, respectively, as frequencies in categories >10 % were small. To identify associations between variables and the outcome variables calf diarrhoea and respiratory tract disease a two-step process was used. First, univariable logistic regression models were applied to determine the associations between the outcome variable and each binary or categorical variable. In the second step, variables with a *P* value ≤0.2 were included in a final multivariable logistic regression model using calf diarrhoea and calf respiratory tract disease incidence (≤10 % vs >10 %) as outcome variable. A backward stepwise elimination of non-significant variables was performed to obtain a minimal model containing only significant variables (*P* < 0.05).

Each significant variable from the final model was subjected to a Mantel–Haenszel analysis to evaluate possible confounding or interaction. Confounding and interaction was monitored by calculating pooled stratum-specific odds ratios (OR). Pooled ORs were compared with the corresponding effect estimates in the whole group (crude OR). When comparing stratified and unstratified effect estimates, an OR difference of >15 % was considered to have a potential confounding effect and consequently the variable was kept in the model [16]. Model fit was evaluated with the Hosmer–Lemeshow test for 10 groups.

## Results and discussion

### Response rate

A total of 1501 breeders answered the questionnaire, resulting in an overall response rate of 14.2 %. According to the previously described exclusion criteria, 214 questionnaires had to be withdrawn, thus, a total of 1287 surveys were used in the final analysis.

The response rate was comparable to and the total number of respondents was greater than in other similarly designed questionnaire based surveys [5, 17, 18]. In other studies on calf management, however, the response rate was greater, achieving 58–73 % [4, 19]. The hyperlink to the questionnaire was sent via email to registered cattle breeder. Due to the nature of this kind of survey and its dissemination, a potential bias may exist, as it cannot be excluded that farms e.g. with severe problems tended not to participate. Furthermore, this survey does not provide any information about calf management practices on not registered farms and farms with no access to the internet. Thus, it can be speculated whether this survey compromises the more professional and modern dairy farms in Austria.

Although the study was not designed as a representative survey, herd characteristics (herd size, breed, milk yield; see chapter general farm characteristics and regional distribution) indicated that this survey gives a good overview on calf management practices on registered dairy breeding farms in Austria.

### General farm characteristics

The median number of dairy cows of farms participating in the study was 20 (interquartile range 13–30). This is similar to the average number of 18 dairy cows per farm on registered farms in Austria [14]. Similar studies were conducted in countries with greater average herd size [7, 11, 13, 20–22]. Data regarding general farm characteristics are presented in Table 1. Distribution of main breeds on registered dairy farms in Austria is 74.5 % Fleckvieh, 13.4 % Brown Swiss, and 10.9 % Holstein Frisian [14] and was similar (*P* > 0.05) in our study. Significant differences between small and large dairy farms were found for all general farm characteristics evaluated (Table 1). In summary, small farms were more often organic producing farms, on small farms cows had access to pasture, and cows were kept tied-up more often than on large farms. Furthermore, farm animals others than cattle were more often kept on small than on large farms. The question regarding “other farm animals than cattle” was included because in a previous study the presence of additional farm animals was associated with diarrhoea in calves [9]. On 54.6 % of the farms the average milk yield was between 6000 and 8000 kg per cow per year. On all registered farms, the average milk yield in 2013 was 7200 kg [14]. Milk yield (given as a categorical variable, see Table 1) differed significantly (*P* < 0.01) between small and large farms. Small farms generally had lower milk yields. Results suggest a lower degree of specialisation of small farms.

**Table 1:** Answers given by the 1287 respondents on general farm characteristics

Variable	Answers	Overall (%)	Small farms (≤20 cows) (%)	Large farms (>20 cows) (%)	*P*
Breed	Fleckvieh	72.9	69.7	76.7	<0.01
	Brown Swiss	14.2	17.3	11.0	
	Holstein–Friesian	7.5	4.8	10.5	
	Others	3.4	4.9	1.5	
	No answer	2.0	3.3	0.3	
Type of farm	Conventional	76.1	70.2	82.7	<0.01
	Organic	23.9	29.8	17.3	
	No answer	0.0	0.0	0.0	
Type of cows’ barn	Free stall	56.4	32.5	81.6	<0.01
	Tie stall	40.2	64.2	15.0	
	No answer	3.4	3.3	3.4	
Access to pasture	No	66.7	56.9	77.6	<0.01
	Yes	31.9	42.0	20.8	
	No answer	1.4	1.1	1.6	
Other farm animals than cattle on farm	No	15.1	12.5	17.3	0.02
	Yes	74.5	76.9	72.0	
	No answer	10.4	10.6	10.7	
Average milk yield per cow per year	<6000 kg	13.2	22.0	3.4	<0.01
	>6–8000 kg	54.6	60.7	48.8	
	>8–10,000 kg	28.8	15.5	42.5	
	>10,000 kg	2.7	1.1	4.5	
	No answer	0.7	0.7	0.8	

Data are given for all farms and for small and large farms, separately. *P*-value presents differences between small and large farms

### Calving and care of the newborn

Details regarding calving management and care of the newborn are presented in Table 2. Calving management and care of the newborn are important for the calves’ health and were therefore evaluated in detail in the present study. Overall, on 47.0 % of the farms a calving pen was available, which is in accordance with other studies [11, 20]. In a German survey on large farms with more than 100 dairy cows, a calving pen was obviously more common with up to 100 % of the farms having such an area [17]. Significant differences were, however, also present in availability of a calving pen between small and large farms of the present study. The use of a calving pen is recommended to minimize stress for the cow and newborn and ensure best hygiene [3, 4]. Nevertheless, in practice, calving pens are often not cleaned and disinfected regularly, or are used also for diseased animals, and might represent a risk factor for spreading infections [9]. This seems to be the case also on the farms of the present study as on farms with a calving pen univariable logistic regression revealed that the odds that farmers reported a diarrhoea incidence of >10 % was greater on farms with a calving pen than on farms without [odds ratio (OR) 1.57, 95 % confidence interval (CI) 1.24–1.98]. This hypothesis, however, was not confirmed in the multivariable logistic regression model, where the reported incidence of diarrhoea was not affected by the presence of a calving pen. As the calving pen might pose a risk factor for the newborn, it is recommended to separate calves from their dam as soon as possible after birth and to house calves individually in a clean area [3]. The vast majority of the farmers (88.5 %) indicated to separate the calf from its dam within 1 h after parturition. This is similar to the findings of Kehoe et al. [20] in Pennsylvania, whereas in studies from Canada, England and Wales, calves were generally separated later from their dam [11, 23].

**Table 2:** Answers given by the 1287 respondents on management regarding calving and care of the newborn calf

Variable	Answers	Overall (%)	Small farms (≤20 cows) (%)	Large farms (>20 cows) (%)	*P*
Presence of calving pen on farm	No	51.1	27.0	68.5	<0.01
	Yes	47.0	70.5	30.4	
	No answer	1.9	2.5	1.1	
Cow calf separation p.n.	Immediately	41.0	46.9	35.8	<0.01
	Within 1 h	47.6	44.7	49.4	
	Within 4 h	3.4	1.8	5.2	
	Later than 4 h	7.8	6.3	9.4	
	No answer	0.2	0.3	0.2	
Time of first colostrum feeding p.n.	Within 4 h	83.7	83.0	84.3	0.36
	4–6 h	13.5	14.6	12.3	
	>6 h	1.1	1.1	1.1	
	No answer	1.7	1.3	2.3	
Quantity of first colostrum fed within the first 6 h p.n.	<2 L	13.3	15.4	11.2	0.11
	2–4 L	71.9	69.9	73.5	
	>4 L	12.7	13.3	12.4	
	No answer	2.1	1.4	2.9	
Checking colostrum quality	No	78.7	80.3	77.7	0.21
	Yes	20.8	19.0	22.0	
	No answer	0.5	0.7	0.3	
If yes, method	Hydrometer	13.5	2.5	23.5	<0.01
	Visual inspection	86.1	97.5	75.0	
	No answer	0.4	0.0	1.5	
Use of an oesophageal feeder for first colostrum	No	63.1	67.5	58.0	<0.01
	Yes	6.0	8.9	3.1	
	If necessary	27.1	17.3	37.6	
	No answer	3.8	6.3	1.3	
Frozen colostrum stocks	No	27.0	35.8	18.6	<0.01
	Yes	72.7	64.2	80.8	
	No answer	0.3	0.0	0.6	
Routine umbilical care	No	26.9	23.8	29.9	0.02
	Yes	69.5	72.3	69.6	
	No answer	3.6	3.9	0.5	
If yes, type of umbilical care	Dipping/spraying^a^	28.4	28.5	28.6	0.64
	Stripping out	17.5	16.5	18.8	
	Combination	54.1	55.0	52.6	
	No answer	0.0	0.0	0.0	

Data are given for all farms and for small and large farms, separately. *P*-value presents differences between small and large farms

*p.n.* post natum

^a^Dipping or spraying with iodine, chlortetracycline or foreshot

Early cow-calf separation is also proposed to ensure an early and targeted colostrum supply [24]. Data concerning colostrum management are summarised in Table 2. Results suggest that farmers are aware of the importance of a timely colostrum supply, as 83.7 % stated to feed first colostrum within 4 h after birth. Although colostrum quality plays an important role in regard to a sufficient immunoglobulin supply to calves, most farmers (97.2 %) did not check first colostrum quality by use of a hydrometer. Regarding time and quantity of first colostrum feeding no difference could be detected between small and large farms. In contrast, frozen colostrum stocks and oesophageal tube feeding of first colostrum were significantly less common on small than on large farms (*P* < 0.01). Although the definition of small and large herds differ between the present study and the study by Kehoe et al. [20], these authors also found that the use of a hydrometer for colostrum quality estimation and the storage of frozen colostrum stocks was more often performed on large farms. Results concerning colostrum management are in accordance with results of a previous case control study performed in Austria [9]. Results of both studies (the present study and [9]) suggest that first colostrum feeding was performed early post natum by most farmers. Answers regarding this question, however, have to be interpreted with care, as farmers may have stated what they know is correct, but may not necessarily represent the true daily management practice. For adequate passive transfer of immunoglobulins (Ig) not only time, but also Ig quantity fed to the calf plays an important role. Although easy, fast, and cheap methods like hydrometers and Brix-refractometers to estimate colostrum quality on farm are available [25], colostrum quality was solely checked on few farms. A reason for this can only be hypothesized. Maybe, in contrast to the knowledge of importance of timely first colostrum feeding, the knowledge on importance of colostrum quality is not widespread.

Routine umbilical care was performed on 69.5 % of the farms. No significant difference was detected between the different routines and the reported incidence of umbilical diseases. This finding, however, has to be interpreted with care as 93.4 % of the respondents answering this question stated that the umbilical disease incidence was ≤10 %.

### Calf housing

Data on calf housing are given in Table 3. Individual calf housing is common in many countries including Austria [11, 19, 26]. No clear tendency was found with regard to the duration of individual housing. Individually calf housing has been suggested with the aim to avoid transmission of pathogens between animals [27]. In epidemiological studies, however, rather group size than grouping itself was associated with an increased risk for calf diseases [4, 28]. Furthermore, it was suggested that social contact provided by group housing of calves increases performance (feed intake, weight gain) and animal welfare [29–31]. Nevertheless, in individually housed calves it is easier to feed calves individually according to their special needs and to control the animals’ health status. The great proportion of farms (84.5 %) housed calves indoors what is similar to studies from Canada and Sweden [11, 19]. Reasons might be exposure to extreme climate conditions in Canada, Sweden, as well as in Austria during winter that prevents farmers from housing calves under outdoor conditions. Furthermore, in Alpine regions large gradients make it often difficult to position outdoor igloos.

**Table 3:** Answers given by the 1287 respondents on calf housing

Variable	Answers	Overall (%)	Small farms (≤20 cows) (%)	Large farms (>20 cows) (%)	*P*
Calves housed p.n.	Individually	88.8	88.0	89.8	0.15
	Individually and in groups	4.7	5.8	3.5	
	In groups	6.3	6.0	6.5	
	No answer	0.2	0.2	0.2	
Calf housing p.n.	Within cows’ barn	46.3	45.2	47.2	0.85
	Own barn for calves and young stock	38.2	38.4	38.1	
	Outdoors	14.2	14.7	13.9	
	In- and outdoors	0.8	0.9	0.6	
	No answer	0.5	0.8	0.2	
If calves are housed individually, duration	1–2 weeks	33.1	33.6	31.7	0.15
	Up to 6 weeks	37.0	33.3	40.9	
	>6 weeks	23.3	22.7	24.7	
	No answer	6.6	10.4	2.7	
Cleaning of calf housings	Regular	61.1	61.8	60.1	0.06
	Infrequently	34.5	33.9	35.4	
	Not at all	2.3	1.6	2.9	
	No answer	2.1	2.7	1.6	
Cleaning	Only dry	23.1	26.5	20.4	<0.01
	With water	10.2	12.0	8.1	
	With high pressure	42.2	39.6	45.1	
	Additional disinfection	19.9	18.1	21.5	
	No answer	4.6	3.8	4.9	

Data are given for all farms and for small and large farms, separately. *P*-value presents differences between small and large farms

*p.n.* post natum

Hygienic measures of calve housings are of importance with regard to reduction of the pathogenic load in the calves’ environment [27, 28]. More than half of the farmers stated to clean the calf housing area regularly. On most of the farms calf housing were not only cleaned dry, but also water and high pressure cleaner were used. An additional disinfection, however, was only performed on 19.9 % of the farms. Nevertheless, no association between hygienic measures and calf diseases were found in present study.

### Calf feeding

On 85.1 % of the farms, calves were fed with whole milk. On 84.1 % (n = 1082) of the farms waste milk (milk from cows with clinical mastitis, high somatic cell counts, or within the withdrawal period after treatment with drugs) was at least fed in exceptional cases to the calves (Table 4). Milk replacer and waste milk were significantly more often fed on large farms (*P* < 0.01). Reasons for whole milk feeding were not asked, but could be due to the fact that it is easy to handle, consists of good balanced nutrients and that milk produced over the quota, can be fed [11]. Feeding waste milk was a common practice on the majority of the farms similar as reported by other authors [11, 13, 23, 32]. Although in a recent study Al Mawly et al. [33] pointed out that waste milk may protect calves from diarrhoea, this practice increases the risk of pathogen transmission and the emerging of antimicrobial resistance in bacteria [34–36].

**Table 4:** Answers given by the 1287 respondents on calf feeding

Variable	Answers	Overall (%)	Small farms (≤20 cows) (%)	Large farms (20 cows (%)	*P*
Type of milk fed	Whole milk	85.1	90.0	79.8	<0.01
	Milk replacer	14.1	9.0	19.6	
	No answer	0.8	1.0	0.6	
Quantity of milk fed daily	Restricted to 12 % of the calves’ BW	58.3	57.2	60.1	<0.01
	Restricted, >12 % of the calves’ BW	28.0	27.6	27.8	
	Ad libitum	11.9	14.3	9.4	
	No answer	1.8	0.9	2.7	
Method of milk feeding	Bucket with artificial teat	75.4	72.9	77.4	<0.01
	Bucket without artificial teat	1.3	1.6	1.1	
	Bucket with, then without artificial teat	18.9	23.9	14.4	
	Automatic milk feeder	2.6	0.0	5.3	
	No answer	1.8	1.6	1.8	
Feeding waste milk to calves	Not at all	14.8	16.5	12.8	0.04
	Yes, to all calves	28.8	26.2	31.7	
	Only to males	30.9	26.9	35.7	
	Only in exceptional cases	24.4	29.0	19.2	
	No answer	1.1	1.4	0.6	
Weaning	<8 weeks	6.5	4.6	8.6	<0.01
	8–9 weeks	16.8	13.9	19.4	
	10–11 weeks	15.4	13.3	18.1	
	12–13 weeks	30.8	33.3	28.1	
	>13 weeks	16.4	23.6	8.7	
	No answer	14.1	11.3	17.1	
Access to water	1–3 weeks	71.5	70.5	72.7	0.26
	4–8 weeks	24.2	24.1	24.2	
	>8 weeks	1.9	2.5	1.3	
	No answer	2.4	2.9	1.8	
Access to hay	1–3 weeks	84.9	84.9	85.3	0.74
	4–8 weeks	13.8	14.5	13.2	
	>8 weeks	0.3	0.3	0.2	
	No answer	1.0	0.3	1.3	
Access to concentrates	1–3 weeks	60.5	52.6	68.8	<0.01
	4–8 weeks	30.2	35.3	25.2	
	>8 weeks	4.9	6.5	3.2	
	No answer	4.4	5.6	2.8	

Data are given for all farms and for small and large farms, separately. *P*-value presents differences between small and large farms

*BW* body weight

On 86.3 % of the farms, milk was fed restricted and on 11.9 % of farms ad libitum. Recent studies state a benefit on growth, health, and performance later in life of feeding larger amounts of milk than the traditional feeding of 10–12 % of the calves’ body weight [37]. This, however, is still not common on dairy farms, neither on farms of the present study nor in other countries that have been surveyed [11, 19]. Weaning took place late (73.7 % of the farmers, answering this question, stated not to wean calves before the 10th week of life) compared with other studies of different countries where calves were usually weaned between week 7 and 10 [11, 13, 19]. Reasons for relatively late weaning on farms were not asked and can only be hypothesised. Advantages of later weaning are higher daily weight gains and a reduced drop in energy intake after weaning [38, 39]. Reported disadvantages, such as higher feeding costs for late weaned calves [38] may be less pronounced in Austria because feeding milk produced over the quota is very common. It will be interesting if late weaning changes with the ending of the milk quota system this year.

Access to concentrates, hay, and water is important for rumen development in calves. On most of the farms calves had free access to hay and concentrates from the first 3 weeks of life (84.9 and 60.5 %, respectively; Table 5). Early concentrate feeding to calves was particularly common on large Austrian farms and is in accordance with studies from other countries [11, 13]. In contrast to the aforementioned studies, in the present study hay was also offered early during the milk feeding period.

**Table 5:** Farmers’ reported proportion of calves suffering from health problems on 1287 Austrian dairy breeding farms

Disease/problem	Reported incidence (%)					No answer
	≤10	>10–25	>25–50	>50–75	>75	
Diarrhoea	51.0	23.4	9.8	4.0	2.6	9.2
Respiratory tract disease	54.7	6.4	1.9	0.5	0.2	36.3
Umbilical disease	57.0	3.5	0.5	0.0	0.1	38.9
Joint problems	47.1	1.3	0.1	0.1	0.0	51.4
Calf mortality^a^	58.0	3.2	0.2	0.0	0.1	38.6

^a^Defined as calves born alive that died within the first 3 weeks of life

### Calf disease

Morbidity and mortality data obtained in the present study have to be interpreted with care. As usually no data on calf diseases exist on farms in Austria [9], we asked the farmers to estimate the proportion of calves suffering from different diseases. Consequently, the results might rather represent sensation of the farmer regarding disease incidence than true incidence. Furthermore, the predetermined classification of incidence was quite rough but considers that on average farms in Austria each case represents approximately 5 %-points. The prevalence of diseases in calves estimated by the farmers is presented in Table 5. Calf diarrhoea was the most prevalent disease. Approximately half of the farmers, however, estimated that the calf diarrhoea incidence on farm was not more than 10 %. This might be comparable to other studies where the reported median herd level incidence for calf diarrhoea was between 7.8 and 10.5 % [4, 7, 40].

Interestingly, in contrast to the question concerning diarrhoea, questions on other diseases were not answered by more than two-thirds of the respondents (Table 5). An explanation for this could be that farmers are more sensitive to calf diarrhoea than to other diseases, particularly as this disease can affect a large number of animals. It can only be speculated whether not answering questions was because farmers were not able to (not knowing) or not wanting to. Not answering questions concerning diseases was not correlated with answers given to any other question (any other dependent variable), but not answering questions to respiratory tract, umbilical, and joint disease, as well as mortality, were correlated with each other (correlation coefficient >0.60). It is probable that certain farmers generally did not want to answer questions on disease prevalence on their farms. Due to the fact that the questionnaires could be answered anonymously and due to the aforementioned knowledge that usually no data on calf disease exist, it is more likely that farmers did not know the incidence of (especially less prevalent) diseases.

Within this study, associations between diarrhoea and respiratory tract disease, respectively, and management on farm were analysed (Table 6). Herd size differed significantly between farms with ≤10 % and >10 % diseased calves, with large farms reporting greater disease incidence than small ones. Associations between herd size and disease were also found in other studies and have been explained by increased stocking density and less time for individual care of calves [8, 9]. Another explanation could be that on farms of different sizes the recognition of diseases differs. Furthermore, other management factors that differ between small and large farms may influence this outcome. Therefore, we tested for confounding of evaluated management factors on the association between herd size and diarrhoea, but could not find such an effect in this study.

**Table 6:** Results of the multivariable logistic regression models with farmer reported incidence of calf diarrhoea and calf respiratory tract diseases, respectively, as outcome variable (≤10 % or >10 %)

Variable	Category	Diarrhoea		OR	95 % CI	Wald *X* ^2^	*P*-value
		≤10 %	>10 %				
Herd size (n cows)	≤20	362	193	1			
	>20	280	306	1.94	1.52–2.47	28.82	<0.001
Feeding waste milk	No, not at all	112	57	1			
	Yes, to all calves	179	165	1.71	1.15–2.54	7.02	0.008
	Only to males	187	179	1.76	1.19–2.61	8.00	0.005
	Only in exceptional cases	169	109	1.30	0.86–1.97	1.57	0.210

Variable		Respiratory tract diseases		OR	95 % CI	Wald *X* ^2^	*P*-value
		≤10 %	>10 %				
Herd size (n cows)	≤20	329	36	1			
	>20	362	77	1.73	1.12–2.65	7.93	0.005
Farm type	Conventional	524	103	1			
	Organic	180	13	0.40	0.21–0.75	6.33	0.014
Calves housed p.n	Individually	630	95	1			
	Individually and/or in groups	73	20	1.97	1.14–3.42	5.81	0.016

Hosmer–Lemeshow for the model diarrhoea and respiratory tract disease, respectively are *P* = 0.99 and 0.92

*OR* odds ratio, *CI* 95 % confidence interval, *p.n.* post natum

In the final model with calf diarrhoea as outcome variable, feeding of waste milk was also associated with the reported incidence of calf diarrhoea. Feeding this kind of milk to all or at least to male calves was associated with higher calf diarrhoea incidence. In contrast, when farmers reported to feed waste milk only in exceptional cases, the reported calf diarrhoea incidence was not higher. As reported in other studies and already mentioned above, feeding waste milk increases the risk of pathogen transmission and the emerging of antimicrobial resistance in bacteria [34–36], that might lead to an increased calf diarrhoea incidence on farm.

On conventional farms an incidence of respiratory tract diseases of >10 % was reported more often than on organic farms. Reasons for this finding can only be hypothesised. It can be speculated whether organic farmers spend more effort in disease prevention as they are limited in using drugs, or if self-assessment of organic and conventional farmers differs. In a study by Bidokhti et al. [41] seroprevalence to bovine coronavirus and bovine respiratory syncytial virus was lower on organic than on conventional farms. The authors stated that possible reasons might be better biosecurity levels on organic farms and limited purchasing of animals to organic farms.

Another factor significantly associated with higher odds for respiratory tract disease on farms was calf housing post natum. On farms where calves were also housed in groups a higher odds for respiratory tract disease incidence >10 % was present than on farms where all calves were housed individually post natum (Table 6). This may suggest that group housing of calves may have a negative influence on the calves` health with regard to respiratory tract diseases. This greater risk might be due to a higher pathogen load and stocking density [27]. No significant interactions were found in the final models.

Significant associations and interactions between calf diarrhoea and calf respiratory tract disease, and between these two diseases and other diseases (umbilical disease, joint problems) were found. This could be because one disease might predispose for other diseases. Another reason could be that pathogens, e.g. Coronavirus, affect several organs [8]. As the time order of the different diseases is unknown, it is not possible to say whether diseases are a risk factor for or a result of diarrhoea and respiratory tract disease, respectively. Furthermore, possibly common herd level factors may increase the animal level predisposition to several diseases. Consequently this study cannot provide cause-effect information.

Several variables that were supposed to be associated with disease in calves did not remain significant in the multivariable model. These variables were e.g. herd characteristics (presence of other farm animals than cattle on farm), colostrum management (time of first colostrum feeding, quantity of colostrum fed, testing for colostrum quality), calf housing (in- vs outdoor housing, hygienic measures), and feeding (milk quantity fed, access to water, hay and concentrates). Some of these factors have already been described as risk factors in previous studies [4, 7, 9, 22, 24, 40, 42]. One reason for these missing significant associations may be that the study was not planned as a risk factor analysis and consequently the study design did not fit. Furthermore, results may be influenced by the fact that disease incidence given by the respondents was mainly based on estimations rather than on true numbers. Finally, some of these factors e.g. colostrum management, were very similar on most farms.

## Conclusion

In conclusion, the results obtained in this study provide data on calf management on dairy breeding farms in Austria. These data may help to further point out areas to be improved on farm, e.g. testing for colostrum quality and prevention strategies for diarrhoea. Furthermore, significant differences could be determined between small and large Austrian farms, suggesting a higher degree of specialisation on large farms. These findings could be important with regard to farm consultancy provided by veterinarians, other specialists and organisations offering advisory services to elaborate prevention strategies to reduce the risk of calf diseases.

## Additional file

Additional file 1. The original online questionnaire in German, distributed to the farmers.

## Abbreviations

OR: odds ratio
95 % CI: 95 % confidence interval
p.n.: post natum
