# Supplementary material for: Results of an online questionnaire to survey calf management practices on dairy cattle breeding farms in Austria and to estimate differences in disease incidences depending on farm structure and management practices
Source: Acta Vet Scand. 2015 Aug 19;57(1):44. doi: 10.1186/s13028-015-0134-y (PMC4539725; doi:10.1186/s13028-015-0134-y)
Supplement: Additional file 1. — The original online questionnaire in German, distributed to the farmers. [file 13028_2015_134_MOESM1_ESM.pdf]

Erhebung zum Kälbermanagement in Milchviehbetrieben

Betriebsdaten 1/6

1. In welchem Bundesland befindet sich Ihr Betrieb?

- Burgenland
- Kärnten
- Niederösterreich
- Oberösterreich
- Salzburg
- Steiermark
- Tirol
- Vorarlberg
- Wien

2.Sind Sie TGD (Tiergesundheitsdienst) Mitglied?

- Ja
- Nein

3. Wie führen Sie Ihren Betrieb?

	Biologisch	Konventionell
Milchvieh		
Mutterkuh		

4. Wieviele Milchkühe/ Mutterkühe befinden sich auf Ihrem Betrieb?

5. Welche Rasse befindet sich vorwiegend in Ihrem Betrieb? vorwiegend...

- Fleckvieh
- Braunvieh
- Schwarzbunte
- Sonstiges:

6. Welche Art der Aufstallung haben Sie bei den laktierenden Kühen? Mehrfach Antworten möglich!

- ☐ Laufstall
- ☐ Anbindehaltung
- ☐ Weidehaltung
- ☐ Sonstiges: ______

7. Nur für Milchviehbetriebe: Wie hoch ist die durchschnittliche Milchleistung Ihrer Herde (305 Tage Standardlaktation)?

- ○ < 6.000 kg
- ○ 6.000-8.000 kg
- ○ 8.000-10.000 kg
- ○ > 10.000 kg

8. Der BVD (Bovine Virus Diarhhoe) Status Ihres Bestandes ist derzeit:

- ○ zertifiziert/frei
- ○ verdächtig
- ○ positiv
- ○ unbekannt

9. Welche und wenn Ja, wieviele der folgenden landwirtschaftliche Nutztiere gibt es am Betrieb?

	1-5 Tiere	6-15 Tiere	16-50 Tiere	mehr als 50 Tiere
Schwein	○	○	○	○
Geflügel	○	○	○	○
Schaf	○	○	○	○
Ziege	○	○	○	○
keine der Genannten	○	○	○	○
keine	○	○	○	○

Abkalbung, Versorgung des Kalbes nach der Geburt 2/6

10. Lassen Sie eine Mutterschutzimpfung gegen Kälberdurchfall durchführen?

- ja
- nein

Zusatzfrage Mutterschutzimpfung

10.a. Wenn ja, mit welchem Impfstoff?

10.b. Seit wann führen Sie die Mutterschutzimpfung durch?

- seit 1 Jahr
- seit 2 Jahren
- seit 3 Jahren
- seit mehr als 3 Jahren

Abkalbung, Versorgung des Kalbes nach der Geburt 2/6

11. Haben Sie einen eigenen Abkalbebereich (z.B. Abkalbebox)?

- ja
- nein

Zusatzfrage Abkalbebox

11.a.Wieviel Prozent Ihrer Kühe kalben in diesem Abkalbebereich? Bitte geschätzte Angabe in Prozent angeben.

Abkalbung, Versorgung des Kalbes nach der Geburt 2/6

12.Für Milchviehbetriebe: Wie lange bleiben die Kälber nach der Geburt gewöhnlich bei der Mutter? Bitte geben Sie die Zeit in Stunden an!

13. Wann wird gewöhnlich das erste Mal Biestmilch verabreicht?

- innerhalb von 4 Stunden nach der Geburt
- innerhalb von 4-6 Stunden nach der Geburt
- später
- gar nicht, Kalb trinkt bei der Kuh

14. Die Biestmilchmenge innerhalb der ersten 12 Stunden beträgt gewöhnlich:

- unter 2 Liter
- 2-4 Liter
- mehr als 4
- unbekannt, Kalb trinkt bei der Kuh

15. Wird die Biestmilchqualität beurteilt?

- ja
- nein

Zusatzfrage Kolostrumqualität

15.a. Wie beurteilen Sie die Kolostrumqualität? Mehrfachantworten möglich!

- Kolostrumspindel/ Kolostrometer/ Biestmilchtester
- Refraktometer
- Augenschein
- Sonstiges:

Abkalbung, Versorgung des Kalbes nach der Geburt 2/6

16. Wird Biestmilch gedrencht?

- nein, nie
- ja, generell
- nur im Notfall

17. Haben Sie Biestmilch für Notfälle eingefroren?

- ja
- nein

18. Wie wird der Nabel des Kalbes nach der Geburt versorgt?

- ○ keine Maßnahmen
- ○ NUR Ausstreichen
- ○ NUR Auftragen von Jod, Blauspray oder Vorlauf
- ○ Ausstreichen UND Auftragen von Jod, Blauspray oder Vorlauf
- ○ Sonstiges: ____

Kälberhaltung 3/6

19. Wie werden die Kälber nach der Trennung von der Mutter aufgestallt?

	Kuhstall	Kälber/Jungtierstall	im Freien
einzeln	○	○	○
direkt in Gruppen	○	○	○
Kalb bleibt bei Mutter	○	○	○

20. Wenn die Kälber Einzelhaltung haben, für wieviele Wochen werden sie einzeln gehalten?

Bitte Angabe in Wochen! ____

21.Für Milchviehbetriebe: Wie wird die Kälberbox/ das Kälberiglu nach der Belegung gereinigt? Alle Schritte die Sie für gewöhnlich durchführen Bitte ankreuzen!

- ☐ Reinigung trocken
- ☐ Reinigung mit kaltem Wasser
- ☐ Reinigung mit heißem Wasser
- ☐ Reinigung mit Hochdruck heiß
- ☐ Reinigung mit Hochdruck kalt
- ☐ abtrocknen lassen
- ☐ Desinfektion
- ☐ Kälberbox/Kälberiglu wird nicht gereinigt

22. Für Milchviehbetriebe: Wird die Kälberbox/ das Kälberiglu nach jeder Belegung (wie in Frage 21 beschrieben) gereinigt?

- ○ ja, nach jeder Belegung
- ○ nein, nicht nach jeder Belegung

Kälberfütterung 4/6

23. Für Milchviehbetriebe: Wie werden die Kälber getränkt? Mehrfachantworten möglich!

- ☐ Saugeimer, von der Geburt bis zum Absetzen
- ☐ Eimer ohne Sauger, von der Geburt bis zum Absetzen
- ☐ Saugeimer, später dann mit einem Eimer ohne Sauger
- ☐ Saugeimer, später dann mit einem Milchautomat (computergesteuert)
- ☐ Sonstiges: ____

24. Für Milchviehbetriebe: Womit werden die Kälber gefüttert? Mehrfachantworten möglich!

- ☐ Milch
- ☐ Milchaustauscher
- ☐ Milch und Milchaustauscher

25.Für Milchviehbetriebe: Wie hoch ist die tägliche Milchmenge für die Kälber?

- ○ rationiert BIS 12% ihres Körpergewichtes
- ○ rationiert MEHR als 12% ihres Körpergewichtes
- ○ sie dürfen trinken so viel sie wollen

26. Wird Sperrmilch (Milch von Kühen mit einer hohen Zellzahl/Euterentzündung, Milch von Kühen, die mit Antibiotika behandelt wurden) an Kälber verfüttert?

- ○ ja, kommt häufig vor. Wenn Sperrmilch anfällt wird sie an männliche Kälber verfüttert
- ○ nein, Sperrmilch wird grundsätzlich nicht verfüttert
- ○ ja, wenn Sperrmilch anfällt kriegen es alle Kälber
- ○ ja, kommt in Ausnahmefällen vor

Kälberfütterung 4/6

27. Ab wann erhalten die Kälber Heu, Wasser und Kraftfutter? Die Zahlen stellen die Lebenswochen dar (1. = 1.Lebenswoche).

	1.	2.-3.	4.-5.	6.-8.	später
Heu	○	○	○	○	○
Wasser	○	○	○	○	○
Kraftfutter	○	○	○	○	○

28. Mit welchem Alter wird der Großteil der Kälber entwöhnt? Bitte geben Sie das Alter in Wochen an! [ ]

Kälbergesundheit 5/6

29. Wie häufig schätzen Sie, dass die folgenden Probleme/ Erkrankungen bei den Kälbern (bis zum Absetzen) in ihrem Betrieb auftreten? Kälbersterblichkeit = wenn Kalb lebend geboren wird und innerhalb der ersten drei Wochen stirbt

	bis 10%	11-25%	26-50%	51-75%	>75%
Durchfall	○	○	○	○	○
Lungenerkrankungen	○	○	○	○	○
Nabelerkrankungen	○	○	○	○	○
Gelenkserkrankungen	○	○	○	○	○
Kälbersterblichkeit	○	○	○	○	○
Gegenseitiges Besaugen	○	○	○	○	○
